# A curated list of InDel markers for rapid genetic mapping in *Arabidopsis thaliana*

**DOI:** 10.5511/plantbiotechnology.26.0420a

**Published:** 2026-06-25

**Authors:** Fahad Mohammed Tonmoy Chowdhury, Koki Mutsuda, Taku Takahashi

**Affiliations:** 1 Graduate School of Environmental, Life, Natural Science and Technology, Okayama University, Okayama 700-8530, Japan

**Keywords:** *Arabidopsis thaliana*, chromosome mapping, EMS-mutagenesis, InDel markers

## Abstract

Identification of novel functional genes through mutant analysis is one of the most powerful approaches in plant science. In the model plant *Arabidopsis thaliana*, this approach typically combines whole-genome sequencing of the mutant with fine mapping using molecular polymorphic markers between two ecotypes to narrow down the genomic region containing the mutant allele. Among PCR-based mapping methods, insertion/deletion (InDel) markers allow for precise and rapid genotyping with relatively simple technology. In this report, we present a set of 49 new InDel markers distributed across the entire genome between Columbia (Col-0) and Landsberg *erecta* (L*er*) accessions, specifically suitable for initial mapping experiments. These markers are designed to detect insertions in Col-0 or deletions in L*er* with a length polymorphism greater than 100 bp and will help facilitate map-based gene cloning.

The isolation and characterization of mutant alleles remain fundamental approaches for elucidating gene function in plants. Since the late 1980s, advances in plant biology have been closely associated with the analysis of *Arabidopsis thaliana* mutants (Koornneef and Meinke 2010; Somerville and Koornneef 2002). The availability of large collections of T-DNA insertion lines from bioresources such as the Arabidopsis Biological Resource Center (ABRC) and the Nottingham Arabidopsis Stock Centre (NASC), together with the rapid development of genome-editing technologies, has enabled efficient generation of targeted loss-of-function mutants. In parallel, the analysis of multiple alleles carrying distinct point mutations, which are usually generated by ethyl methanesulfonate (EMS)-mutagenesis, has gained renewed importance, as such allelic series often provide deeper insights into protein function than complete gene knockouts alone.

In mutant-based studies, considerable attention is typically devoted to the design of screening strategies for identifying mutants under appropriate experimental conditions. However, the subsequent identification of the causative locus on the chromosome is equally critical for timely and reliable downstream analyses. Currently, one of the most widely used approaches for identifying causative mutations in *Arabidopsis* is bulked segregant analysis coupled with whole-genome sequencing (BSA-seq) (Hartwig et al. 2012; Schneeberger et al. 2009; Uchida et al. 2011). In this method, for instance, mutants in the Columbia (Col-0) background are crossed with the Landsberg *erecta* (L*er*) wild type, genomic DNA from multiple F_2_ individuals displaying the mutant phenotype is pooled, and whole-genome sequencing is performed to identify candidate regions enriched for Col-0-derived alleles and harboring point mutations (Uchida et al. 2014).

While BSA-seq has proven to be highly effective, it is often complemented by classical map-based cloning to increase mapping resolution and confidence in the identification of the causative gene. In addition, BSA-seq analyses of dominant mutations typically require whole-genome sequencing of the original mutant line to serve as a reference when wild-type-like F_2_ individuals are used, which may reduce cost efficiency. Furthermore, map-based cloning remains particularly advantageous for the analysis of structural variations, such as large deletions, insertions, or inversions, especially those induced by mutagens other than EMS, for which standard SNP-based approaches may be less informative (Yu et al. 2021). Given these considerations, mapping strategies based on easily scorable polymorphic markers continue to represent a practical and valuable tool for *Arabidopsis* genetics. While there are several papers on polymorphic markers useful for map-based cloning in *Arabidopsis* (Hou et al. 2010; Pacurar et al. 2012; Zhang et al. 2007), we previously reported InDel polymorphism markers between the Col-0 and L*er* accessions and demonstrated their utility for genetic mapping (Tanaka et al. 2020). Here we further refined this marker set by selecting polymorphisms that allow unambiguous scoring and developed a streamlined panel of markers that enables rapid identification of the chromosome carrying a mutation within a single day.

For *Arabidopsis* genomic DNA extraction, tissue samples were ground with micro pestle in a microtube with 120 µl DNA extraction buffer that contains 0.2 M Tris-HCl (pH 8.0), 0.25 M NaCl, 25 mM EDTA and 0.5% (w/v) SDS. After the tube was vortexed for 15 s and centrifuged at 15,000 rpm at room temperature for 10 min, the supernatant of 80 µl was transferred to a new tube containing 80 µl isopropanol and mixed. The tube was centrifuged at 15,000 rpm for 10 min, rinsed with 100 µl of 70% ethanol, dried under vacuum for 10 min and dissolved in 50 µl TE buffer. After centrifugation at 15,000 rpm for 1 min, the supernatant was used as a template DNA for PCR. For genotyping, PCR was performed using Ex Premier DNA Polymerase (Takara Bio, Kyoto, Japan) with 45 cycles of denaturation at 94°C for 10 s, annealing at 55°C for 10 s and extension at 68°C for 60 s. The reaction mixture consisted with 3 µl of 2× Premix, 1 µl of 5 µM each of forward and reverse primers, and 2 µl of the DNA sample. The PCR products were separated on 1.0% (w/v) agarose gels in TAE buffer and visualized with ethidium bromide.

When designing InDel polymorphism markers between the Col-0 and L*er* accessions, we selected polymorphisms in which the insertion/deletion size difference was 100 bp or greater and in which the Col-0 allele was longer. This strategy was adopted to identify the chromosome harboring a mutation rapidly and unambiguously using the smallest possible number of markers. Because such polymorphisms are frequently found in transposon-related sequences, primer design was preceded by BLAST searches to avoid regions with similar sequences and thereby prevent nonspecific amplification. As a result, we were able to establish 49 new polymorphic markers across the entire genome (Figure 1). The PCR primer sequences for each marker are shown in Table 1. Among these, we propose using a total of 16 markers as the initial set for screening: four markers on chromosome 1, which is the longest chromosome, and three markers each on chromosomes 2 through 5 (Figure 1). Gel electrophoresis results for wild-type Col-0, wild-type L*er*, and F1 hybrid plants are presented in Figure 2. We confirmed that none of the markers showed biased amplification favoring one allele over the other.

**Figure 1:**
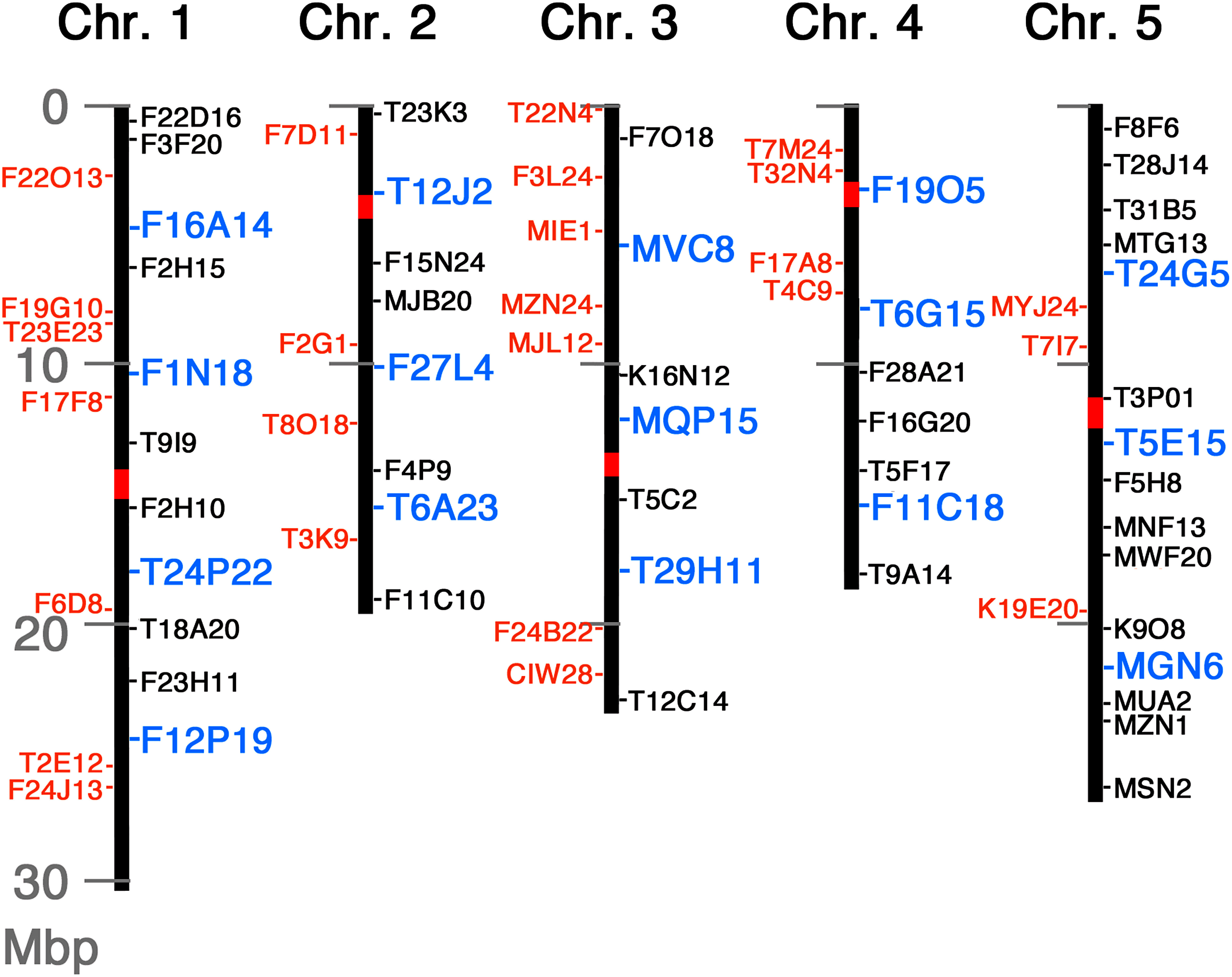
Figure 1. Linkage map of InDel markers on *Arabidopsis* chromosomes. The positions of the 16 markers recommended as a first choice for one-day rough mapping are shown in blue, while those of additional markers developed in this study are shown in black. The positions of previously reported InDel markers are indicated in orange in the left side of each chromosome. Centromeric regions are shown as red boxes.

**Table 1:** Table 1. A comprehensive list of 73 InDel (Col-0>L*er*) markers useful for Col-0/L*er* genotyping.

Chr.	Marker/BAC	Position	Primer sequence		ca. DNA length (bp)		Closest gene
	Name*,**	(Mbp)	Forward	Reverse	Col-0	Ler	ID
1	F22D16	0.675	CACGGGCTTGTTCATATGTC	GTAGAGGTGCAGTGGATCTTC	580	350	At1g02970
	F3F20	1.705	CCGCCTCCGAGTCTTTTTAC	GAAAAATGAAGGCCACGAATG	1700	420	At1g05680
	F22O13*	2.826	GTGTTGGGGAGAGCTTATAG	TCCACTTCCAACCATCAGAG	460	210	At1g08830
	**F16A14**	**4.742**	**TTGGAGTGGGCCAACGCTTG**	**GTGCAACGGTTATTCAAATTACG**	**1500**	**480**	**At1g13850**
	F2H15	6.173	CTTCGGCTACTACTTCACTAG	GTGGAAAATTCAGCAATGCAATG	1000	700	At1g17940
	F19G10**	8.141	ATGTCACCGTGAACGACATC	TGCGAGTTAAGACCTAGGAG	470	320	At1g22990
	T23E23*	8.494	AAGGTCTTGTAGCGATCTAG	AACCCAACTGGCTCATTTTG	540	420	At1g24000
	**F1N18**	**10.441**	**CTGCTGGAGATGTCTTTGATG**	**CTCACCTTAGGAGAAGTACAG**	**1080**	**770**	**At1g29820**
	F17F8*	11.016	GGAAGAGGATTGACTCAAAG	CTACCGCTAGGACTTTCATG	460	360	At1g30930
	T9I1	12.973	GTTAAGAAGGCCTAGAACTG	GGGGATGTTCTTACTCATATAG	1000	780	At1g35340
	F2H10	16.149	CTGTTTGGGTTTATGAAAGAGTG	CATGAGGCTACTCTCAACCTCT	1160	730	At1g43000
	**T24P22**	**18.058**	**CACGGAATTTGTTTTTCCGAAG**	**CTTGACTTGAGGAACAAACAAC**	**1530**	**440**	**At1g48830**
	F6D8*	19.621	GAGACACAGAGGAAGTGAAG	CTGACCAGCAAATTCTCAAG	640	540	At1g52690
	T18A20	20.106	AGTGTTGTCTCGTAAAAGTATG	GGATATGGTGCACCGGAGAG	1000	700	At1g53850
	F23H11	22.069	GGGGATGTTCTTACTCATATAG	AGGGGTGTGCATGGATACTC	1100	500	At1g59950
	**F12P19**	**24.535**	**ATCGAGACGAGAGCCTTGTG**	**GTGGAACTACACGATACAAAG**	**1320**	**800**	**At1g65930**
	T2E12**	25.66	CGACTAGCCAGTCCGATACA	CGTTTTGGGAGCCACGTTTC	530	280	At1g68450
	F24J13*	26.624	GCTACCCTTCAAGAGATGAG	TCGTAGAGTTGCAGCAAAAG	600	500	At1g70610
2	T23K3	0.416	GGTGGAGAAGAGCGGTAATG	GCAGAGGAAAGAAACAAAAGAG	1400	900	At2g01905
	F7D11	1.614	AGCGAACTTCGTTGATGTTC	CAATGTATATGCTCTTCTAGAG	670	570	At2g04622
	**T12J2**	**3.594**	**CCCACAAGGCAGCATCAATG**	**GCTAGGGAACTGCAGAGC**	**1700**	**1190**	**At2g07810**
	F15N24	6.022	GAGGCTGATATATACGGTATC	CCAGAGCTTCATGTTGTTAG	550	450	At2g14210
	MJB20	7.585	CGTTTCTCTGGAGCTGCCTG	GGCAATCTGGTTTATGGTTTAG	740	560	At2g17470
	F2G1**	9.268	CGTCGTCGGAAGTTTCAGAG	GAATAAGAAGAACACATGCGTC	410	250	At2g21680
	**F27L4**	**10.075**	**CCCCTCTGTTCAGTCCTTTG**	**ACTCAGGGAGATCCCGAAAG**	**1500**	**600**	**At2g23700**
	T8O18**	12.273	GATATGGATGTAACGACCCAA	CAGCTTCGAGTGGATTCTAC	700	360	At2g28625
	F4P9	14.117	CTGTGGGGTTGTCTGTAAAG	CCTCTTGCAAACTAATACTCTC	840	640	At2g33310
	**T6A23**	**16.15**	**GGTGATGATTTTGAGTGCTC**	**CATGTATGGTAATTGCATCAG**	**1490**	**870**	**At2g38610**
	T3K9*	17.107	TCATCGGAAGGAGCATTATG	AGGATGTTCCAGAGAGAATG	470	360	At2g40990
	F11C10	19.071	CCTAAGTTTCCACAGGATAAAG	AGCACAACGAGAAGAGTGAG	1500	1000	At2g46460
3	T22N4*	0.13	TGACTGTTTGACTCCAAGTG	GTTACGAACCTCTGGTATTG	2040	400	At3g01345
	F7O18	1.266	TCTGGATCTCAAGGGCTCTG	CCTTTTGGCGTCTACTCTTG	1000	450	At3g04670
	F3L24*	2.849	TGAGCAATGATGGTTAGCAG	GAACGTAACTGCTTACGTAG	770	470	At3g09270
	MIE1**	4.873	CTAAGTTCTTCCACCATCTG	CAAGGAGCATCTAGCCAGAG	450	330	At3g14520
	**MVC8**	**5.377**	**GGGAACGAAGACAGAACCTG**	**TGAGTGGGATTTGGTACAAG**	**1410**	**460**	**At3g15900**
	MZN24*	7.665	ATCCGAACCGAAATCAACTG	GACTGAACGAGAGGAACATG	1250	500	At3g21750
	MJL12*	9.194	GGAGGCTAGAGACTCATATG	AGGGGATATTCGACTGAGAG	650	490	At3g25240
	K16N12	10.354	GAGAAGAACCCTAGATCGTAAC	CGGAACCTTATTTTGACAGTG	1340	550	At3g27900
	**MQP15**	**12.145**	**TATCTGGTCCCCTCGATATG**	**GGTGACTCATCTCCTGAAAG**	**1210**	**570**	**At3g30416**
	T5C2	15.309	AGGAAGAGCGAATCAGATAG	TTCCCTTAGCTTGGCAGAAG	1000	750	At3g43357
	**T29H11**	**17.884**	**GGTGGTTTTGGCTAACTTAG**	**TACGTAACGAGGGTCCACTC**	**1400**	**880**	**At3g48208**
	F24B22**	20.093	CTGGGAACAAAGGTGTCATC	CAAGGTCTCCAGAACACAAAC	430	330	At3g54280
	CIW28*	21.869	GAGCACAAGTCTCTTACAAG	CCCTAAGTTTCACAAAGAATG	630	450	At3g59140
	T12C14	23.085	CTTTCAGCCCTTTGTATTCTC	CACGATGCAGTTCTCTTGTC	1200	600	At3g62380
4	T7M24*	1.788	TTTGGCGCTGTTGCCAATTG	TAATGCGCGAGGTGGATATG	1350	710	At4g03826
	T32N4*	2.549	CTCAAGGTCGACATGATAATG	GTATAACGCGGGTCAATCTC	1180	900	At4g04985
	**F19O5**	**3.321**	**ATTTCCCTTTGGTACACCAG**	**GCGTGCCACGGTGATGTTTG**	**1590**	**980**	**At4g06526**
	F17A8*	6.109	TGCTCGAGAGACTTTTCGAG	CATAGACAGCCACACCAATG	1280	560	At4g09670
	T4C9**	7.299	CAAAGGTTTCGTGTCGGAGC	CGTTGACGGGATACTCGGTG	650	280	At4g12270
	**T6G15**	**7.923**	**AGAATCCATCGACACTGAAG**	**AGGACTTCGTCGGATTTGTG**	**1060**	**800**	**At4g13615**
	F28A21	10.316	AAGGATTGCCGCAACGCAAG	GAAGGTCGAATTTGATGGATG	1200	950	At4g18780
	F16G20	12.22	ATGTAAACTCAGTCTAAACGAG	ACTAGAAGTCAGCATATATCAG	1300	400	At4g23380
	T5F17	14.134	ACCAGTTTCTGATGGTTCTG	AGCAACAACGATAGTGCTTG	800	670	At4g28610
	**F11C18**	**15.397**	**TCCCTTCAAGTTCGATTCAG**	**TGTGTTATAGGTATATGCCATG**	**1220**	**680**	**At4g31830**
	T9A14	18.087	GAGACTGCACATAAGGTGTAG	TCTACAGTCGGCAGTGCTTC	900	650	At4g38740
5	F8F6	1.026	GAGACAAAGAGACGACCGAG	GGACAAAACCTTTGGGTGTG	1500	400	At5g03840
	T28J14	2.243	ACAACCATCCAAGTTTCCAG	TTGCTCTAGGTTGTCTGGTG	1000	800	At5g07200
	T31B5	4.242	CTGCGCGCTCATATTAGAAG	GGTTCGTGCTCGCTCCATTG	1000	600	At5g13260
	MTG13	5.442	CGTTTGTTGTTCCTAACCAG	GATTCGTCAGCTTACATGGTG	1300	500	At5g16610
	**T24G5**	**6.46**	**AATCATAGCCGCATCATCTG**	**ACGGCCTGTAAACAAAGTAG**	**1480**	**900**	**At5g19210**
	MYJ24**	7.753	CTAATCCCAAGCTGAATCAC	TGACAGAGAATCCGACTGTG	570	290	At5g23100
	T7I7*	9.271	TGGCACCAAGAAGCAACTAG	TCCTAACTATCAACCAACTTG	650	500	At5g26594
	T3P01	11.29	GGTTATGGTTCTTATATTCGTTTG	GTGTTTCTGCATCGGCTTTG	1000	750	At5g29624
	**T5E15**	**13.08**	**ATTCTCTCATCGCCTTCCAC**	**GATAGAGAGAGTGATAGCTC**	**910**	**200**	**At5g34871**
	F5H8	14.52	CTATCACCATGTATGGATTCAG	GAAGACGCTCAAAACCTTATG	1200	650	At5g36870
	MNF13	16.301	CAAAATTGGAGTTTGAtGCCTC	GTGTGGTTTTAGCTTCCGTC	1000	600	At5g40750
	MWF20	17.393	CCTGGACATTGAATGTTAGTG	CCTTACCGGCTAACCGGATTG	700	530	At5g43340
	K19E20**	19.83	GACAAGAACCACATGAGAGC	GTTATGTGTACACTTCAGGTC	620	340	At5g48900
	K9O8	20.296	GGGTTTGTGAACAATGGATG	AGGTAGTTCCACCATTCAAG	880	630	At5g49900
	**MGN6**	**21.84**	**TCACGTGGGTCAGCCTTCAG**	**CACACATGTCAAAAGTTTACGAG**	**1270**	**550**	**At5g53780**
	MUA2	23.33	CGGTCATAAGCTTATAGCATG	CGCTTTGGTCAAATGTACAC	600	400	At5g57620
	MZN1	23.75	CAGGTGTGGCTGCGTACATC	TGGGTTGGGTCTTGTACCATG	1040	890	At5g58820
	MSN2	26.61	GTGGGACTGAAAAGCCCAAC	CGCCACTGCGTTTGTGACTG	880	670	At5g66660

Sixteen markers recommended as a first choice for initial mapping are in bold letters and shaded. Asterisks indicate markers reported by * Tanaka et al. (2020), and ** Zhang et al. (2007), respectively.

**Figure 2:**
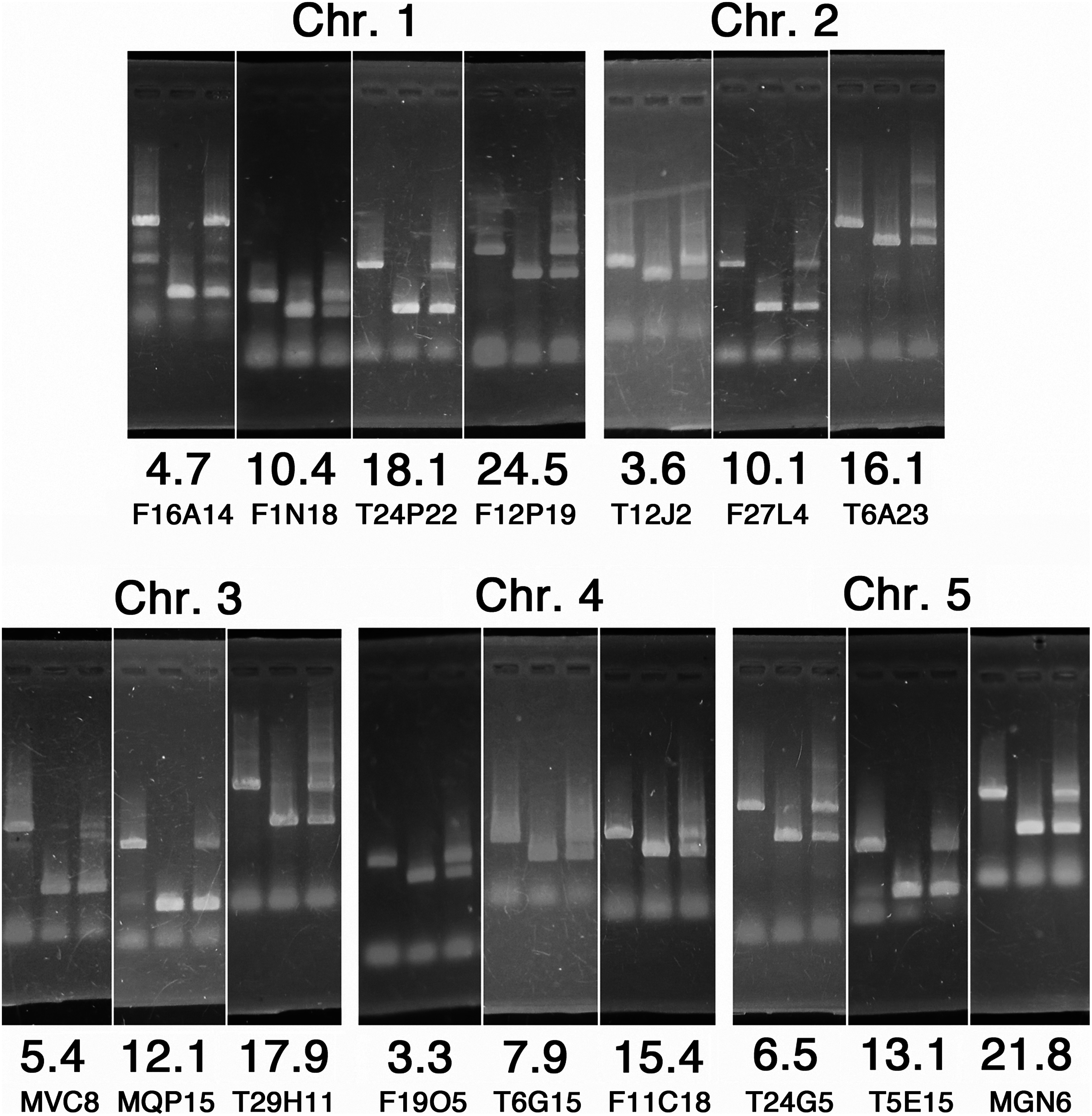
Figure 2. Band patterns of the 16 InDel markers listed in Table 1. PCR-amplified DNA fragments were separated on 1% agarose gels. In each panel, samples from Col-0, L*er*, Col-0/L*er* F1 progeny, and a mixture of these three genotypes are shown from left to right.

PCR amplification efficiency or nonspecific amplification sometimes depends on the commercial source of Taq DNA polymerase and primer redesign may be required in certain cases. In this study, as in previous work (Tanaka et al. 2020), primers were designed to be 20–22 nucleotides in length, with a GC content of approximately 9–11 nucleotides. The 3′ end was designed to terminate in G or C to enhance specificity, while avoiding an excessive concentration of GC residues at the 3′ terminus to prevent mispriming. For reference in primer redesign, the DNA sequences of the Col-0 genomic regions containing the primers for each of the 16 markers, as well as the corresponding regions deleted in L*er*, are shown in Figure S1. We further examined whether genotyping by PCR using these markers would be feasible in mixed samples containing two individuals. Genomic DNA was mixed in combinations of three Col-0 individuals and one L*er* individual, as well as the reciprocal combination, followed by PCR amplification. The results showed that, for all primer sets tested, amplification of the less abundant genotype could be detected. A representative example is shown in Figure S2.

The workflow for chromosome mapping using these markers is illustrated in Figure 3. Assuming that a mutation is obtained in the Col-0 background, the mutant is crossed with wild-type L*er*, and the dominance, semi-dominance, or recessiveness of the mutation is first assessed in the F1 generation. In the F2 generation, individuals are selected according to the inheritance pattern: wild-type phenotype for dominant mutations, mutant or wild-type phenotypes for semi-dominant mutations, and mutant phenotypes for recessive mutations. DNA is then extracted from the selected individuals and subjected to PCR analysis. Using two individuals as one sample, PCR is performed with the 16 polymorphic markers shown in Figure 2, followed by a single round of gel electrophoresis. Empirically, the experiment can be completed within one day by analyzing DNA from eight individuals (four samples) using these 16 markers and allow identification, with very high frequency, of genomic regions in which homozygous polymorphisms are enriched.

**Figure 3:**
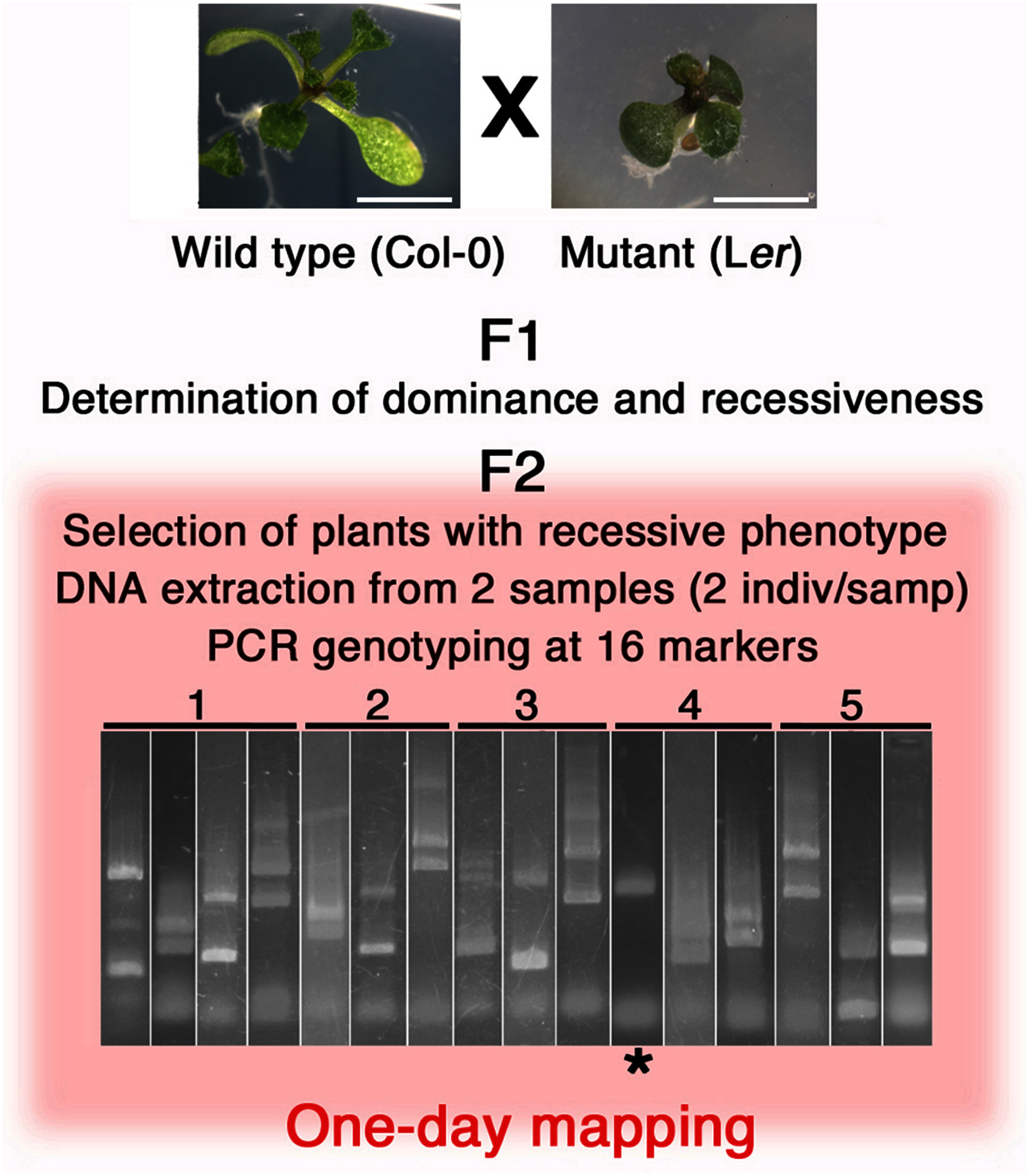
Figure 3. Workflow for one-day rapid mapping of an *Arabidopsis* mutation. The number above a gel image indicates the chromosome number. An asterisk denotes a marker showing a single band, suggesting linkage between the marker and the mutation locus. “One day” refers to conducting an experiment of a scale involving DNA extraction from 2 to 4 samples, followed by PCR using the 16 markers and gel electrophoresis. Bars in seedling photos=5 mm.

As a supplement, the use of visible markers in combination with PCR-based chromosome mapping is expected to further reduce the labor required for mapping. When a mutant is isolated in the Col-0 background and crossed with wild type in the L*er* background, linkage to the phenotype of *erecta* (*er*, At2g26330) which is characterized by reduced internode elongation in the inflorescence and round leaves with short petioles, can be effectively utilized. The use of L*er* lines carrying multiple visible mutations may further accelerate the mapping process. For example, a L*er* line harboring multiple mutations, including *chlorina1-1* (*ch1-1*, At1g44446), which confers pale green leaves; *glabrous1* (*gl1*, At3g27920), which causes a trichome-deficient phenotype; *eceriferum2* (*cer2*, At4g24510), which displays glossy stems and leaves; and *transparent testa3* (*tt3*, At5g42800), which produces yellow seed coats, is available from the ABRC.

Previously reported polymorphic markers, when subjected to PCR using bulked samples from multiple individuals, often exhibited a problem in which one of the bands became difficult to distinguish. This issue was particularly evident for markers with small differences in fragment length and for those with amplification bias. Here we carefully selected the markers, together with previously reported InDel markers (Col-0>L*er*) that showed clear and easily distinguishable genotypes (Tanaka et al. 2020; Zhang et al. 2007). When whole-genome sequencing is outsourced to a commercial DNA sequencing service, further fine mapping can be conducted while awaiting the sequencing results, making it highly likely that the causal point mutation can be identified immediately upon completion of sequencing. In our experience, for three different dominant mutations, we successfully narrowed down their chromosome positions with the use of these markers and identified nucleotide substitutions by mixing genomic DNA from mutants into a single sample for whole-genome sequencing and using the wild-type genome from the ABRC database as the reference sequence (Mutsuda et al. 2025). In contrast, when performing the same analysis using BSA-seq, it is necessary to determine the mutant genome sequence as an additional reference. Taken together, the polymorphic marker information presented here is expected to be highly useful for future identification of causal genes in mutants, offering clear advantages in terms of not requiring special equipment, cost-effectiveness, speed, and accuracy.

## Abbreviations

BSA-seq: bulked segregant analysis coupled with whole-genome sequencing
Col-0: Columbia-0
EMS: ethyl methanesulfonate
InDel: insertion/deletion
L*er*: Landsberg *erecta*

## Description of supplementary files

Supplementary Figure S1. Genomic DNA sequences of the Col-0 wild type amplified by PCR using primers for the 16 InDel markers shown in Figure 2.

Supplementary Figure S2. Representative band patterns of PCR amplification using mixed DNA samples from multiple individuals.
