# Supplementary material for: A curated list of InDel markers for rapid genetic mapping in Arabidopsis thaliana
Source: Plant Biotechnol (Tokyo). 2026 Jun 25;43(2):227–31. doi: 10.5511/plantbiotechnology.26.0420a (PMC13324382; doi:10.5511/plantbiotechnology.26.0420a)
Supplement: Supplementary Data [file plantbiotechnology-43-2-26.0420a-s001.pdf]

Supplementary Figure S1.

Chr.1 - 4.742 Mb

ttggagtgggccaacgcttgctataatagttatatgtttaatacagttcaaattcagcaaatGGGGATGTAGCTC
AAATGGTAGAGCGCTCGCTTTGCATGCGAGAGGCACGGGGTTCGATCCCCCGCATCTCCAattttgaaacttttt
ttttgattaaaggtttttcagacatctgcactccaggtttcgctgttgtgtctttggattcacgtcttgacattg
cctccatgcatatgcgagatcattaggttttgcagatgtcactttactcaatgtttttgtgcagttttcttcgac
ttaaagactttttttatagctcccatttctatttcctatgtaggcttctggcggacattaattagggtataacat
aaccaagtcttttctaagtcataatcctaataataaagcatatcaagttttaggttaacaaattaaagcattgga
aaacataaccagttgagctgacatatacaggtggtggatttggctttgcgtttcttcttccctaaagacttgcgc
ttgtagtggaggaatcgcaaatttgaactacttgatcggagctgagtgaagtgaaaaacattcattcaacatcta
tacactaatacacatatatatgagcaataacaaccatcaccatccatataaagagattatcaagaagagaaatgg
acacctacccaagatgtgtggaggtagtagtaacatcgtcaggcttggacaatgttttcagcagagtaaggccta
aaaggcttagggattcttctagaaggtgcatattatagtagtcacatctaagagaacctgaataatgctccatac
accatttacacacaccgatacgatacgccgatggaagtctccaaaattgacgacacctcgttcgacgcttcccca
tgattcttgtcttgtgaacttttggtgtgcgtgcctcttgaacaatttggggaggccagaaacccaagtttgggc
ctcttatattaattcaaggttttacatctgactcactctatcgagaagatcgactctgctagtgctagggtttcc
gagaggcagtgaaaatcatatgattagatagagtgggcaataagtttatgtaaatacatattattatatacttgc
tacaacatctaaataagcaataaacaagcaataGGGGATGTAGCTCAAATGGTAGAGCGCTCGCTTTGCATGCGA
GAGGCACGGGGTTCGATCCCCCGCATCTCCAattgttatccttctttttttaataacggtattattattatcatt
ctataatttatattaccagccgaaatatctttacaactttttaagttatgttctgtcatttctgttctctacaga
aacaattttgttttgattcattagaggaatgaatgaatatacgagagagatgattcttaatgaataattacgaaa
cgaacaaaaaataaaactgagaacaattcttgtttgcaaacagaaaaaatgtaaagaatgt***cgtaatttgaataa***
ccgttgcac

Chr.1 - 10.441 Mb

CTGCTGGAGATGTCTTTGATGCTGATTATATTCTCATCCAGTAAacaaacttggaatctattttctgttaagata
ctactaaattcagagagatgtcaatacaatgttgttgcataaaagataaaagtagtctgaatcactgagagaaca
aagctattaagtcgaggtctctgttaaattttatgtgccagcatctatttcgctaaaatttcgaccaaattatt
cgtcttccatattcttccaattgtacatttcatgtctgaaatagtaaaagagtgagtaacgaagaattattttgg
ttacaattattcttaaaccaaaatgaggaatgagtgtagaaaaggtgttatgaagcatgaacatttcctcaaaga
gataagacaaaaaaaaaactagttgttcttcacaacgactgtcttaatgaattacataactgttgtaactaattg
actttgatgtttggtataacaATGATCAGCTCCTTCCATAATCTCCAGCTTGTGGTTTGGTATGAGCTTTGCAAA
CTCCTTGGCATCTTCCACCGGTATTACCTCGTCAGTCGAGCCATGAACCGTCAAGACCCTGCCATTACAACCAAT
CATGgtttgtttgtatcccaaacatggatatgagttagttaaagggaaatcagcgaatctcacctgcattatttg
tcaatcttgaggcaagcttcatgcatatcagtgtttaacctttccattaagctctcctcggtaacacgaaacccg
gagtttcctgtaaaagacccgcaccttgataaagacactactactagagtactgatatgtgacagtagatgaaga
acaaagagcgatgaccttctttaatatcgatgaacccttgtttctttattctttggtgttacatgtcacatttca
taactttacggttaactatgtaaaaatgtaaaacaataaggagcaaatttatgaaaacgtttacgcgcgtctctc
aaagtcaaagctaaacaatttgcttgtaaagtattcataatttgcgaactctcgagtgttggatcagccatctgg
agaaagagaata***ctgtacttctcctaaggtgag***

Chr.1 - 18.058 Mb

cacggaatttgtttttccgaagtttcttttgtaaactttttttcttaaacaatcatgtacgtagtatatttttaa
ttaatgtgtgggtgacaaaaaaaaaatgtaatttcaaacaaaaaaagttagaatttggtgttatttagttaataa
cacttcataaaaattctttaaacatattgctaagaataaaacatgggataatggtaaataaaatgaaaattttaa
aatgagaaattaggttggatatttaaaatgaggaaaatgccactttgtttagataaacatttttttaaaattaaa
aagatgcttttcaattgttcatataaccctcaaattttattagcaatttttagtatcgattcagtaaatatgtaa
tgtatagtagtagagacatgaattaatgtcatgtttcatgaaaagagaattaacaaaaataatacaaacactaga
tcatttttaaatttctcaacgtaaatatgatattttataaaaattgatgatgaccatacgtccataaatattaat
aatgtagGTGGGATCCCGATGATCATGCCATGGATGGACTACCAGATTATTTGAAATCTGTAGTTAAATTTGTAT
TCAATACCTTCCAAGAGTTTGAAAGAAAGgttggagaagagaactaatagcaaaactcaatgatttttttttac
gatcatatatagtagtaggtcactatatagtaaatgtttacacttttgagccgttattgcagTGCAAGAGATCGT
TGAGGATCAACCTTCAAGTCGCTAAATGGGTAAAAGCGGGTCACTTGCCTAGCTTTGATGAGTACCTAGATGTTG
CTGGACTCGAGCTCGCTATATCTTTTACCTTCGCAGGTATTTTGATGGGTATGGAAAATGTCTGCAAGCCGGAAG
CTTATGAGTGGTTGAAATCTAGAGACAAACTCGTTAGAGGAGTAATTACAAAAGTCCGATTGTTGAATGATATCT
TTGGCTATGAGgtagggaagattttcccatttttagttaatttttcgataatcatatgacgttgttctttcataa
tgaaacttaacaagtgacatgattagGATGACATGAGGAGAGGATATGTCACGAACTCGATCAACTGTTATAAGA
AGCAATACGGAGTTACGGAAGAAGAGGCTATTAGAAAGCTTCATCAAATTGTTGCAGACGGTGAAAAGATGATGA
ATGAGGAGTTTTTGAAGCCAATCAATGTGCCATACCAGGTTCCAAAGGTAGTCATTCTCGACACTTTACGTGCGG
CGAATGTCAGCTATGAAAAGGATGATGAATTTACCCGTCCTGGTGAACATCTCAAGAACTGCATCACGTCAATTT
ATTTTGATCTTTAGaactttggtttatcgaacttacttttgagttttagtttgtaactgggtactcgctctacaa
aactatatctcgtctcttgtcttgtgttttaagatacctgaactgaccatgtctttacaaaacaagtctttgttt
ttttttcaatt***gttgtttgttcctcaagtcaag***

Chr.1 - 24.535 Mb

atcgagacgagagccttgtgttgcttatttcaaaagctaaactctaaactctacaaacaaaactttaaaccatac
atttttttaagtaaattataaaccaaaatggtgtatttacataatgtgtagtctatgtttatgtggaacaatacgt
ttaattcaatatcacatgtctatttatcgagtaatatttgtttgtttttttaataaacaaatcagacatgcggaaa
aaatctatagtttttttttcattagtcatgcgagtttaatcaattttgttaaagatatataaagaaatgaaaggg
atataaaatttactttacttagaaaaaagagaatgagaaaaataaatgatgacatgaagtaattaatttatcttg
tctcactaaggaaaaatatgtccattttcaaaaattaataaagaaatttgatatagttatataattttttcacta
aagcattgtcatttctccatattcctctatgaataatggaggaagaaaatagtatactgaaagagtttttttaaa
aatagtatattcatcctttaccagacaaaggtaatatttctagctttgtgaagaccttaaaccacttatcgctat
atattgtggtagttgatataatcttgttttgtttcggtttaaattgctattgaacaaataacccatacataaata
tataaatgttatgcgaaaaaatcaattataagtatacatgggaatgaaatcaaatctgcttatatgtgactttgt
atttaactagtcggagacaagtcagagaagtgctttctaccatgtctcttaatgtttgtcaattaggtcatacca
caatgtcttgttttcattatccatgtccatgtggcatttataatagctgaataaatcataaataaatccaatttt
atgggcatggatatggctataagtaacttagaagtatcgaaagtccaaaccagatctcattatgatatattgttt
aagattttttgtgaaacaattgtattttagtaatcaactacttaacctcattgatgcatctcgctaccatgcagat
tatggtactaacacaaatgactacactaaatttacttgtcaactaaagcaattcctaaagatagtaaagagagaa
ctatatacaaaatgtttccagacaaagacatgcagtgtactgatttgagttacttttttaagacttcctttagttg
ttgactaggacatatttttagctaaaagatatttctttcaatactaaaaatattttgcccatattttttagtattt
aacgtaaacatgaaatcttacacta***ctttgtatcgtgtagttccac***

Chr.2 - 3.594 Mb

cccacaaggcagcatcaatgagtgaatccctgagtaaaaaaagaagtggaggaaatattagatcttataaactaa
tggttaaaacgaaaaaaatttacacatgaactaaaggtgaaaataaaaattacccaagatctttaaactaaagat
taagcaatagtgatgatctccttaactttattcatcccaataatctctctaataatgtttgcaaattagaaaatt
gcataaagaaataacttacgtttgtatgaagagaaaaaactgtagataataaaacaaattatagcgatggcaatg
atgtacccataatataatatcctacttatatcctactttttactttttacagaagcttgtatcctacttccatgt
ttcaaaataaaatataaaccttgtcactaacagtttacatcaagttataaaagaatacattgatttagacaaata
tccaactaaaataaatttaattttataaaaaaaaaatatccacaaggtcgtttgattctaatccaagtcattagt
agtttgtaacgacaaagattagagaggcggtttagtatatgtgatactgtttcatatagcaaagaatgaaatata
gaaaattgaatcaaacgaacctcttataataccaaactttaaagatggtaaaaactataatgtagtagtacaac
agagctctacattagtctcatcagttctccttcttctaagtaaatcttaacataggtcatgatataatagatttt
agtgagacttcagaaaaatccacaaaattgtaccttatcatctacaagaagaagattcaatccagtgaattccct
tatttatcctttttttggggaaatccccaagggcacttctcaccttgatttacatcgatccgttactagagtt
atatgagagataggtgtaatattgagtgacataattggagattggaatttggaatatgaacctacaaataaaaag
ttgtttttcttaaatagagaattttatgagcaagagaaacttatgcaatttagataaagagaatagaccatgtgg
gtcagtggttcgacacagtaatgccaaaaattctaaaatttgcagacacaataacaccaaatcacttgagagaag
ataattgaactataacaaaatttacaaacctgagaaatacgaaaacttgtaaacttgcatagaggaagagacatg
tgattgatgataaaccctaaaagtaaatcatgtaacgacatcatacccaaagatgaattggaaaccaagaaggga
tgatgtgaagaggagaggcaaagtgagaggcggaagaaataatgatactttttcggttaaagaggaaaggttgaa
agtgaggaataatagggccatgtttgtttgatcatttggaatttgcatccaaatgatccatccaaataatccatt
tgaatgattcatcttaatgatgtttgtttgattatttgctaatcatttgctaattcatctaaatgcatcatttag
atggagctttgtttgtttgatcatttggatgaatcattcacattctcatatatatatatataaatgactaaaatg
ccctctgttaagtaacaaataatcaataactacattctcatattaatcaaagtagagtcttaaaaagagagttct
ctgtttccattttctaagttccttcttat***gctctgcagttccctagc***

Chr.2 - 10.075 Mb

cccctctgttcagtcctttgtttcaaggtttttggttttagatattattcttgaaaccatcgatggatatttt
tactacctaatagttgggtgtaggcctgggagttcggttagtcggtttaatctggttggtttaatcggtttttcg
ggtttttcggattttaaaaattctgccgaattggaccgaattaaatttcggttcgggtcggttcggtactcggtt
ttttcggttcggttacagggattaaaatcaattccgaactaaaccgacccttttctaaatcggttaattcaaac
cgaattagaccaaaattaaaaattatgggctcaattattaaatttacaattataaaccaacaataataaattaat
aatggggtaaatctaataaaagtccaagcccaaaataaacaacatgcccaagcccatctgaaatctaggtttttg
acaagtgaagaatgatgacggtgacggagacgccgttcactcatgcgcgtccgactttcaaagggaaagagagaa
taattagcggcttgactagatctgtaattcctacatagtgcagatgtctatgtttgccaccaccggcaactagac
gagagatttgatgtcgcggcggtgagaaaaaaaaaaaatggattgatgccagagagtgttggtttcacagcagta
aacgaatatgataagctgagaacgaaaacgaagaagactgaagaaggagaagaagatcaaagaaggatcttgagg
agaagagtttaagattagaaaaggaaggagacgacatgaattgttgacatagatctagggtttcttctattttat
agggtgtacctctaaaccgagaaaaccgatcggtttagcttgccgaaccgaaccgaaccgtaatccgaatcacaa
tattatttttgccgaacggtttgccttctctttatctctctgcgccgagaaccgaaacattcggtttcctcgggt
ttagccgaaatcccaggcatagttgggtgactgaaacttggaatttagtagaccacattatttatgtcgttgtgt
tatcatatcattttaaattcggtaaaatttgtgtatgagttattgacataaacaaaagtaaatgaaatgagagat
cactcgggcgagagtttaaggagaagaaaatcagctaggaaattttgcttagtaatatatatcgttactataaca
aataaattttaaaaaataagagaatcgattttgtatgtacaaacatcatcgcggattggtaaccaaaaaacggca
tgcaatttttcctctatttctatttctactctacactcaccacacaagacatccacctatacactaaaccaataa
tacattacaaagaaacagagtccttgcaaataaaaacccttcccaagacaatcacataagaaaattTCATCTAAC
AAGTTCTCTGGCTATCAAATACCGAAACACGAAATTATGAGGCGTCCATTCGACAATGCT***CTTTCGGGATCTCCC
TGAGT***

Chr.2 - 16.150 Mb

ggtgatgattttgagtgctcttattatgctcatggtcttccaagtcccccaaatgtaacgcacacaattaaacca
ttttacgtgtacagaattttataagacaaagcaatttatgaatattttggaagagaaaaaatattaataaaaagg
aatttaatgaaaaaaactctcaactttgtcctgatccattttaaaccctaaactatgttttagtaaaacaaat
cctaaactaaaacctgttaataaacttaacttcacagtaattaaatattaatgagatattaatttttaaaaaga
aaacaatgagacatttgtatttagcgcaaaaaccacgtgatgttctttaaaactgttttgaatttattacaaaaa
aacattctaaataattttgaattgcttaattagtttggattttaaaagctgaaagaaagacacaataacttaaaa
taatccaaagagtcatgcttaaataaatccaaccaacataactatcgaagctgttgcaacacaacaatatttgtc
acaaaaaaaaaacaatcgtttcagatggacaagatattctaaagtaaagacttgtcaagcattaactttgaagag
aagattgtattggctgagtgatagggttctcttcatccattgttttctgtttttttccttggtcgtccttgaggtt
tcttgggtggttttggcattgctttacacttgcatgaacgtttgttgtgtccgacttgttgacaattgctgcaag
tctaaatatgcaaagattaaatttaataatttgaaaatgaagatcttgtgaaacataattcattgttctctattt
taccatttaaactttttcacgtgtataaagaacttttgaggtggacgatcttcagttggatcacggattctgtc
attgttctttggtcgattttggaaattaatatcccattaatatttaattactatggggttaagtttattaacag
gttttagttcagagtttgttttactaaaaacatagttcagaatttaaaaatagatctgaccaaagctgacggttt
ttttcattaaattcccttaataaaatggttgcaaataagtttattgaccctagtaaatcagtaacggtttttcat
aatcaactttaaagtctttaagccattttctttcgtttagtcaatgccctatcattgaatattatctttgttttc
tttttcattattgtaattttcttttatgtatagtagtccaataatttttaatattcagccattgtttagtttcc
acatgaaacacacaatttgtttataatcttgtttttctttcccctttacatagaacttcttatgatgatttttat
gagtttgtgaaattgcataccttgcacattcgtagacctagtcagaatttttgttgttgttgtagtatctttatt
gtaaaaagaaatggttttcttaatgtaactatcgatcaaaa***ctgatgcaattaccatacatg***

Chr.3 - 5.377 Mb

GGGAACGAAGACAGAACCTGACATctctctatcttttctctctaattctgtcgtattttgtggtcacacaggctt
gtgaatggcgtgaacagaggataagagagagagacacgacgcttaccaaaaacacatctcacgttttgcgtcgat
tcttgaaactttagaacgcaaacgctccccacaaacccacgtttgcgttttcactcactttcataacaaactaaa
gattctgaggaacaaatacgtaaggaaaaaaacctgaaaaaaatcttagtttatttttatttgcctgtttaatatc
tatattatataatttgtccaaataatacttaggttaattattttctctttaaaaatcttcattttgtattaattt
ggcaaaatcagacatccaacttaacggatgttaacttttaacagatgtttatagttaaaatttaactcatgata
tttacaaatatatatacaatctattttctatattctataaatatgttaccttagtctagcggttaaacacagatt
taatttctgatatacctgggttcgaaactttcttacgacgtttatagtctcttttttttttccttcttcttctt
tctcggaatttaaagatttcatccagacaaaattaagttcatcttcttcttattggtacgatgtttttaacttct
caagagagacaaataacacgaattgatgaagctcggcttaacttcgacaaagctactttaatttttttcgaagg
ttctgtaaaaaaaaattcagatccaaacagcgatttcaacatgacgatttcttttaggtttattaatttgattagc
ttttgttattgtgtggtcaatttgattggggattcaaacctaaatttgagaaagcaaaaaaaaaacgaatccaga
tttggcgaagaagatgaagtcccaaaaaaaaaaaaacgaatacaaagacgtaagcgagattcgtacccaggttaaa
catgaattgaatatcatatttgccttgactaagataacttaattgtaaaattttggaaatagattgtatatata
tttgtaaatacgatgggttaaactttaactacagttagaaagtctgttaaaaagttaacagcagttaagttggat
gtctgattttatcaaattaaacaaaaatgaaggtttttaaagagaaaaataattaacctaaatattatttgggca
aattatataatgcaggtattaaacggccaaataaaaaataaatgagagtttttttcaggttttttcccccaagatt
tttataatacacctaaaaagaaccagcgatctttgtcttcgctaaaacaaaccaaatctaggaaacttgtttaat
aataacatgcttaaacttgtttatttttaaacaagtgtt***cttgtaccaaatcccactca***

Chr.3 - 12.145 Mb

tatctggtcccctcgatatgattttttgtaggcaagccactacgagaactttacttatcataatgaggcccaataa
gcagaggcccactgaagacctatattactgctgacttgttagatagcctatgagcagagttgagatcaagagaca
tccaaatatagttttggaatgtataaaaagttggatatcatttcagtatataacagaattttattttatttttac
cattgcttacatattgtttttgtttcaaattataaatgctataaagtattacagaagaaagaccttatttaaatg
ttagtttttgattttgctgacttaactttactttcccttggtcccaccaagcaaacccatctttcgtctctgctc
acttataatattaaccgtcgacgatttgtcactaagtataatggaggagggacctgcaaagttgctcacgccttc
gCTAATAACCTCCCAAAACAATGCTCCCGCGGCCGAGCCGCCTTTTTGCGTAGATATCGTAAATGATGTCATATG
TTGTGGTGAACACGTCGTCTCTATTAGCCAGAGTGTAGCGTGGTGTGTCTGATCCTAACCCGAATTCTGCTAGAA
TTACAGGTTTTTTAAGATTGTTTTGCGCATCTTCGATGTGGCCTTCAAGCCATCTTTTTATGAAGGCCAATCGAG
AGTTCTGATCCAACCTTTGAAACctgcgcaaaaccaaaaagaaaaagctcaatggtagtcgatttgtaaaccta
ataacatatttttttatatatagaatcgatgttattgtttatagctctgacgtatatacgtttaggataaatgaat
atatgtagttttacatactgatatggatataaaacaaaattttcaattgctacttttgcaaagaaaaactagctc
ggaagataaatcccaaatgatagcatgggtggattaaagcaaaataatttttttcctaggttgatgtgttaaaca
aggaacaaaaaaagagagcatgaatactatttctgaaagattgttttgtctaaatggatgtacagtataaaactt
agtgtgaatatgtcagttgatcatacCATAAGTCACTTCCACAGTGCATCGATGCGAAATCAATGGCATCGATAT
TGTGATTAGCTATGAAATCAGCTCCAACTGTGTTGGCTGAAACTGGATTTAGTGAATTCTTT***CTTTCAGGAGATG
AGTCACC***

Chr.3 - 17.884 Mb

GGTGGTTTTGGCTAACTTAGTGCATCAGTTTGATTGGACACTACCGGAGGAATCTACAGAATATCAGACTGATGT
CGCTGAATCAACCGGTATGGCGGTTCATCGCATGTTCCCACTCTTCGCCATGACTACTTGAtttagacattacag
catatagatatgctttgttagaagcgtacaatacgcatgtcagtggtaaagagatcttaagatttgagttcttaa
gtaaactatagactaatagtttgactcaaaaaaaagtaaccacactaattaagtaagccaagcggcataatcagt
tccctcgcaattatcaacggaatataacagatcgatgtttccttctcaattaggctcttagtataagttccactg
tagacataaatgagtaaatataagatatagatagagaccatgtatgcatgcaaaacttaaaaaaaacaaacagaa
atcaattatatatttttaattagggtggatcagattaaacttctggaactttaggatatcctttcgatcttgta
aaatttttataagatttttatcttcctattttaagactatatattaaaaagtggaaaagattggattatatcaac
cctaattcaccgtctaatttgttttcctctttatgccattacatcatctactacttgattaaggcattatataca
ttaaaatgatttatattatgttactagatcagaacccatttgatacatgggttaatcaataaatgttttttata
aagtttaataaggtttttttgcagttttatctaaatattaataggctatgaaaatataatatggtgaagatttta
agattttatgaaaatataatagacttctggaatttgatgctttagttttattacaaaagcttgtctagaagactt
gaagtattagacaatataattgaggaaaatgaaagacagtaaaacgcttttgttgatgaaatcgaaacaattacc
aaatttccgaggctgaaaagagatatgtcaatgccttagattagaggtgagttactttttggtgtgttttcacat
atttcttcaaagttattagctttgatatttaaatattctacatttacaaacaaataggtggtagaatcatacct a
aaatttaaaaagttattaatatacaaataaggactatatattttaggagataaatagttttcttggaccttgtag
ctgtctaaaatctagcaaaataggtattaaaaaaagtaaaagatttcttttttgtttggtcaacttacttgtatt
gtggagagattttttacctttactatataggatttgagagaatatatatttaatgcaaatttactttcctactat
acttgggcaaaacagaagaaaattt***gagtggaccctcgttacgta***

Chr.4 - 3.321 Mb

atttccctttggtacaccagtaattttgcagaagcaacgcaaaacatcctatttccggcatttcctagtgttcgt
cttcccactatggtcatcccactatggttacctataagacaagtagaatcatgagtaatctttgattactcaatg
agctgggtctatgcacaataaatctctagggtttaatcaactccataagtaaacttagccaccagagatcacat
aacacgtaataaacgtattatcatatacttataataatacaagaatatgcagccaactagacacatgaatgaacc
ttgggcccaacatactgccaaccttaccaaaaaggtgcagcacaacatcagcacaaactcccgcatacgtgtata
cgtgttctccacacgtatgctatcacacacacgtacgtgcacacccacaccacacgtacatacacaactgtgaac
acccgagctatagcaacaaaggcttataaccagaacatcactttaatgcatcgtcatgcattacccaacatacgc
ttcgagtcttgtgccatcatgtaagtgaccacttaggatcaagacttactcacacaatcacaacacaaaaaggaa
ggcagcaacttactctaagcatgatggtatcgggtaaacccctgaataccacttagacacaataacctcatgcaa
tactaaatataagtagtttaccacaccccgaacaatatagacaatcaagtaccaagtcacgtattaatatgtac
ataatataataaggacttattcacaacctcataacatatctcatataagtttaacaatctaaaaaggcaagtaag
ccctcgaatcatatctcaattcatcaagtaaagtaagtcaagtccgaattcacttacctaatcatgcaccagtcc
atcccattccttagtgatatcaagttcttcaaatgcccatattcactttaagattcaagttctaacttaatccaa
attccgaatagttaagtctaactaagtaagcatgcatatgatctaaagactagctctagaaaacatgcaaacaaa
cagacaattcgaccacaactcacggattcggtagttcggaaggtttctgggtctcgggcgaatctcctcgatcat
ggtcgacaatcctttactcactttcgagttctctcaaccacaagcaacaattttctagtcacgggcagagtttcc
tcgaccacgggcgatgattctctgaattcggtcgagtttcttgattttggtcgagttattggtcggacagcctta
cagtctctctctctcgccttcaaaactaaaattaggcgaagtgagctctatttatatcccgcccaaggtcggcca
gactgcactgcctgctgccttatgtccggacagctgtgacgccaacaagcgatagttgtctcgggtgatggcgtg
gcgactggtggcgatgggtgtcactccaccacctcagcagcttcggctccaagacccagccaacttcggctccaa
gattgattacttgagccgcaaggcaccgcgtgtgaggtgtcctacccatgtcggttccgcggacggcgggg***caaa
catcaccgtggcacgc***

Chr.4 - 7.923 Mb

AGAATCCATCGACACTGAAGGAATGATGGAACACTACACTAACTGATCTCAGCCAGCTTATAGTTTTTTTTTTCA
TTTTggtttgcgttttgttatacgggttttgttatcagaaagaaaaaacagagagaagatttcattatgtctta
gttgctaattgctatgtttcctataggtagtgttctccaaaacaagagataattctccctgttgttattgctttt
gatgcctagatacacaatttattcaacattttctaatcatcttagaatttcaaatatacactaacatgttaccgt
tgtaaaacaccagagtcttgttcttcttcatgttcttctcctaagcatttgtcttcctctgaaaaaagaaaaaat
atagaatataccgggtctttataagtctgatgttcggaagattcatttagctcatttgcatcctcaatctttgtt
tttctcatctagtacgtcagatgtcaagagccattgaataaactcagatcttttcgtctgctctccctgatcttg
tgttgagacagatgaggaagattaaccaaactatcccattcattgttaatctggtaccttctgaagaagataaca
acatctttacccagagttcaatcaagtatattttgcaaaaactagcctagtataagtcacgtgtaagatgcatgt
agaacacagaattttttttgttttgatatcaatgttaagtatttataccattttcgaagttttgacagaagttat
aaagaatacaagtaatggaaaaataaatctacagaaagcacaaacaaaaacaaacagtaatggaggaaatcttta
accaagagcggaaacaagaaaagaaacaccgattccgagaagctaaactcgaaaatttcagtgatttccacaact
cgaaaataccaaggagctggaatctcatcttacttgccacgagcaaaccttagcagaggtccgtcctcaaaacct
ctcgagtaaggcacattaacttctcttcaaactaacgcaaaacgtaaagataatagagaacttaaa***cacaaatcc
gacgaagtcct***

Chr.4 - 15.397 Mb

tcccttcaagttcgattcagtaacaatctcacatgtatgtatgcttaaggttaaagtttcaaatccatgcttaaa
tggttgaaaataaataataggaaaaatgtcaaaaaatgcgctaattttcaaatttgggacgaaaaagcatgaat
tttcgaaatgtcatttaaatcttaaagtttgggttgacttcttaaaaaaatgtctaagttttgttgaccttgcat
tttaaagcgtgccgtgaaatttccgttgagctagaaccttagatcactggatctgtagtcaagcattaaaccgtt
gagctactcacgcttttggtgtttacttaagctaatagatatatatattcgtacatgtaattaattgaaaaacac
aaacctaaaagctccattattgcctgcgtaataaacagcgtgaacaagtccagaaaataactctgaatcagatgt
aatcaagagttcacaatacttgaaatcgacaacattaaacggatctccaattgtataaatccgagaacggtactc
tccccaaacaaaccatcattggacatagacgagccacacgatctcaacctcacgctcatatttgacgtgattcca
cgccacctgctcacgctgttttcttctcaggcaacaatggagttctattagtttcaactagttacatatatataa
gctcaatcaatgttgttacaagtttacagtagctccatggtttacagtagctccgtggtttacagtagctccatg
gttgaaggcttaattacagctgcagggaatcgtcggtttgacactaggttgtgtccgattttcttcttatttta
atttttaaaacgacgtcgttttgaactgttaacggaaagttgactccgttaattttctgttaacgcaaatttcac
ggcatgccttaaatgaccacgtcaacgaaacttagacatttttttaataagccaactcaaactttgagatttaaat
gacatttcgaaagttcatgctttttttcgtcccaaatttgaaagttggcgcatttttcaacatttttcccaataaa
taatacatcaaatgacaaaaataaattgactggaagactgcaaatggtatcaaatgaatatgtaccctaaacaga
gaattgatggaatatactgtagtcaatatagtattgcgtttgtatttttatgtcatagagaacataaaaag***catg***
gcatatacctataacaca

Chr.5 - 6.460 Mb

AATCATAGCCGCATCATCTGGCTCAATGAAAACCAATGAAGTAGCCAAAATATGTTTTGATGGTATCAAAGCAGG
AAAATTCACAGTGACATGCCATTTTATTGGCTTCTTACTATCTATTGCTAGCACCGGCATGTCCCCTCAGGGATC
TTTTTGGCTTGCACTCACTGAAGTTATGTTTGGTGGTCTAATAAGACTCGCCAGCTTGGTTTTCCAATGGCAATG
GTACAAAACCATAGAAAAGTGGAGCCAAAGAAATAAAAAAGAAGTAAATAGTAAGTTAGCTTAGgttatcaaagt
aattgtttttggtatgaaccattatctctttggaacaggtttatgattttgtagaggttgataagaaaagattca
tgacatctactatgatcagtcttttgtatagtgtacactgaacaggttcatattttctagttccaacttcggatt
ttgttgacaatttaaatagggaaaaatgttatttaatacctgaactttcaaaaagtggccaaattaaccgtgaac
tcttaaaataaccgttttatacctcaacaaaaagttgacttctaatttaatctataagttatcgttgacctaacc
aaatcgactcaccattaacagtcatgaacaactttcctaactgcgtaactaacagctgttttcgtccttaaacca
acgataacgactgttagcccatgttttgtaatggctgttaggagcgttgttaagaagtgttaacggtgaatcgat
ttgccctgatcaacgataacttataggttaaattagaagtcaactttttgttaaggtataaaacggctatttcaa
gagttcacggttaatttgaccactttttaaaagttcaggtattaaataacatttttctcaatttaaatatcaata
aatttggctcctccaataaaaaagaaagcactaaaatttgacaaagtaaaaacagagtttttatctgtaagtaa
gaaaaaacaaattcattatccttcgtttccgatttccacttcactccggaggtgctcaaactcaaaaATGGCGTC
GCAGCTTCTTAATGTTCCTCATCTCGCTTTCTTCCCTAAAATCTCATATGCTTCCGTCTTCTCAACACTAAAGCC
CAGTTTCTTCCACTCTACTTCCACTAGAAGAGCTCTCAAATCATCCCCCAGTTCCCGGATTATCAACCTCCAAGC
CGTCGCCGAAACTAGCTCTGAAATCGAAAGCAATAGTGTCACTGAAACAACTGTGCCATTGACTCTACGTCAAAT
CTGCCAAGGTTTTGTGCCCGAGCATATCTTGCACAGgcaagcttcgtttctctcctttttatttctgtagaatttt
gaattttgaattttgacaagtactaaaaattcgaattttgtgaattgatgcagAATGGAGGAGATTGGATTTGTC
TTCCCCACGGATATTCAAAGAGAAGCTCTTC***CTACTTTGTTTACAGGCCGT***

Chr.5 - 13.080 Mb

attctctcatcgccttccacaatatctcaactctcaaatatttttttgtttagttttaagtttttttggttgttg
ttatgagttaaaaaaaatatcattttaaaatcttagttcaaaattttaattttagacaatgaaccttacattgtt
ttttctttgtttttaagaaatatatatatatccattatctatttttattaccatcttataaataatcattctatc
atcctattccactatattcatgtatatatatatatatatatatatatatatatatcttatgttgttatatggg
taaaacacaataaattaaagattaactcccacaatcttctggaataggatgatatacacaataaatttaaaaagt
tttaataaaattttgaatgaataaaatatttattgaaaattttgagatatatgttttatatgtctattaatagtt
aataatttaaattccttgaattttataatattaccatgacaaatagtttaatttaaatacataaataaaaaataa
ctctaatacaacttttaggtttcacaacatttatcaaaagaatagttttttttgtttataactctctctgtagttt
tagatattttggatgactgcgcatttttacgtaagacgttttgattgaatctcatgaaataaattattgtatgga
gaataactttaacgactttctcattttggtatccgcaaattcattattgtttttctttttatctttgcttcattag
ttagtatagctcttctaaaaaaagattctatacaaaattatattcataagcttatctatactttttttttaacaac
taatatttttctcacattcttttcaatatgtatatgaatttttgtcttcacaatttttctcaga***gagctatcact***
ctctctatc

Chr.5 - 21.840 Mb

tcacgtgggtcagccttcagccttgtttattagcaagattttacaagatattgttcgtttgaccacttaaccact
tctttaatccggtcgcattagttaccatagtgacaaaaagcccaagtagtataaggtttcaagcccaattatatt
gttttagcaggccaggcttacTTAGATTTAAAACCAAATTTGTCTTCATTTTTTCTCTGAACGAGAATCGAAAGC
ATtttttatacttgattcattacccattatttactaaattatttacaaaatttgccattaatattatatataaat
atttcaacaacataaaaaataaaaagcaaattctaatttaaataattaaaaatgaaattccaatcaaaacaactt
aataattaagcaagtaacatttcggaacttaatatttaactaccttcatttttttttatataaacataaaattata
tgttaacaactatattgatatcaattaatatatggattataataatttatatgccaaaaaatcctagttcaaata
aaatagttatgtatattacattacataatacacaagtaaatttcagatttaatataataatccaataaattttgt
aatacactcaatatatttacttaataattaatttgtcctgcatatagtaaatgtaaactatctaatgtattttag
tgtatttttataaagtgtctacgcagcatttaaaatctcaccaacttatttattctcgattgtaaataattggta
gtgtaatattttgtgtaagaaccagaaacttcgttttagtctatacataattcactctaaatcctaaaatcatta
atgtacacaaaataattggggcaacgagcacataaaaatactaacaaggaaactatatgtaccgaaatctttaac
atatttcacgttaaaactaacaaaatatgtttttttattttttttttgtcgtcaactaacaaaatatgttagataat
tcgaaaatgatatcaaatataatccaaaataaagtaatgagaaaatacttcaaaaagtatagataaatataatca
tctaaaaagtaaacatataacaaattaaagaatgagattaaaactatataactatgaattgtaaaaaaagaaaca
aataaacattgagagtaatatatatactttttaaataaatattacttggttataagaaattaaaagtgtacttat
ataagtttaagaccctaacaacgcaacacgtgtagtaattataa***ctcgtaaacttttgacatgtgtg***

Supplementary Figure S1. Genomic DNA sequences of the Col-0 wild type amplified by PCR using primers for the 16 InDel markers shown in Figure 2. Sequences deleted in the L*er* accession are boxed. Primer sequences are shown in bold italic letters. Transcribed sequences are shown in uppercase letters.

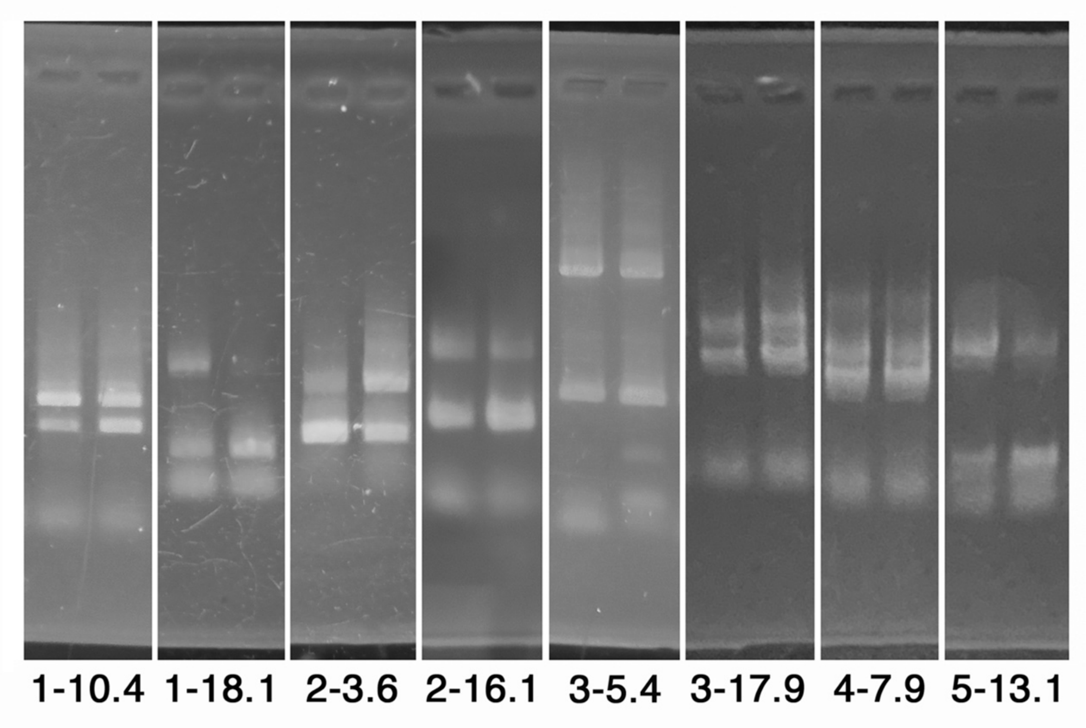

Supplementary Figure S2. Representative band patterns of PCR amplification using mixed DNA samples from multiple individuals. PCR-amplified DNA fragments were separated on 1% agarose gels. In each panel, the left lane shows a mixture of DNA from three Col-0 individuals and one L*er* individual, whereas the right lane shows the reciprocal combination (three L*er* individuals and one Col-0 individual).
